# Optimized Illumina PCR-free library preparation for bacterial whole genome sequencing and analysis of factors influencing de novo assembly

**DOI:** 10.1186/s13104-016-2072-9

**Published:** 2016-05-12

**Authors:** Christopher Huptas, Siegfried Scherer, Mareike Wenning

**Affiliations:** Lehrstuhl für Mikrobielle Ökologie, Zentralinstitut für Ernährungs-und Lebensmittelforschung (ZIEL), Wissenschaftszentrum Weihenstephan, Technische Universität München, Weihenstephaner Berg 3, 85354 Freising, Germany

**Keywords:** Illumina, Next generation sequencing, PCR-free sample preparation, Assembler, Read depth, Read size, Average library insert size, Assembly performance, Genomic GC content, Sequencing quality

## Abstract

**Background:**

Next-generation sequencing (NGS) technology has paved the way for rapid and cost-efficient de novo sequencing of bacterial genomes. In particular, the introduction of PCR-free library preparation procedures (LPPs) lead to major improvements as PCR bias is largely reduced. However, in order to facilitate the assembly of Illumina paired-end sequence data and to enhance assembly performance, an increase of insert sizes to facilitate the repeat bridging and resolution capabilities of current state of the art assembly tools is needed. In addition, information concerning the relationships between genomic GC content, library insert size and sequencing quality as well as the influence of library insert size, read length and sequencing depth on assembly performance would be helpful to specifically target sequencing projects.

**Results:**

Optimized DNA fragmentation settings and fine-tuned resuspension buffer to bead buffer ratios during fragment size selection were integrated in the Illumina TruSeq^®^ DNA PCR-free LPP in order to produce sequencing libraries varying in average insert size for bacterial genomes within a range of 35.4–73.0 % GC content. The modified protocol consumes only half of the reagents per sample, thus doubling the number of preparations possible with a kit. Examination of different libraries revealed that sequencing quality decreases with increased genomic GC content and with larger insert sizes. The estimation of assembly performance using assembly metrics like corrected NG50 and NGA50 showed that libraries with larger insert sizes can result in substantial assembly improvements as long as appropriate assembly tools are chosen. However, such improvements seem to be limited to genomes with a low to medium GC content. A positive trend between read length and assembly performance was observed while sequencing depth is less important, provided a minimum coverage is reached.

**Conclusions:**

Based on the optimized protocol developed, sequencing libraries with flexible insert sizes and lower reagent costs can be generated. Furthermore, increased knowledge about the interplay of sequencing quality, insert size, genomic GC content, read length, sequencing depth and the assembler used will help molecular biologists to set up an optimal experimental and analytical framework with respect to Illumina next-generation sequencing of bacterial genomes.

**Electronic supplementary material:**

The online version of this article (doi:10.1186/s13104-016-2072-9) contains supplementary material, which is available to authorized users.

## Background

Next-generation sequencing (NGS) has revolutionized genomic and genetic research [1]. Today, Illumina’s concept of massive parallel sequencing-by-synthesis using a fluorescent reversible terminator chemistry [2] represents one of the predominant NGS technologies [3].

During the last years, many efforts have been undertaken to improve existing library preparation procedures (LPPs) for paired-end genome sequencing [4–7]. Currently, the commercially offered Illumina TruSeq^®^ DNA PCR-free LPP represents one of the most widely used solutions for the generation of paired-end genome sequencing libraries. It includes genomic DNA shearing by adaptive focused acoustics, which leads to random fragmentation of DNA in contrast to the more directed fragmentation via enzymatic digestion. Unbiased shearing of DNA and the abolition of PCR significantly reduces the unevenness of sequencing depth across sequenced genomes [5, 8]. Additionally, the use of magnetic beads for DNA clean-up and size selection is much less prone to contamination compared to traditional gel-based systems. One of the main drawbacks is the need for very high amounts of starting material (1–4 µg total DNA) that precludes the application of PCR-free protocols for samples where the increase of DNA amounts via cell division is impossible. Consequently, the main application will be bacterial strains growing well under laboratory conditions.

In its current design the original LPP is directed to the generation of libraries with only two rather short average fragment lengths (350 or 550 bps). This is unfavourable for eukaryotic genomes, since sequencing projects could make use of multiple sequencing libraries with varying insert sizes to enhance assembly quality [9, 10]. On the contrary, it was one of the main findings of GAGE-B (genome assembly gold-standard evaluation for bacteria) that remarkably good assemblies are possible with a strategy using just one Illumina library, but high-coverage in case of bacterial or other small-sized genomes [11]. To further elevate assembly performance, it seems advisable to increase insert sizes of bacterial sequencing libraries for better exploitation of the repeat bridging and resolution capabilities of current state of the art assembly tools [12].

The generation of genome data based solely on a single Illumina library limits the parameter space in which enhancements of assembly performance can be achieved. Main factors relevant in this context are read quality [13], sequencing depth [14–16], read length [9], assembly software (including parameter tuning) [11, 13, 17, 18] and the repetitiveness of the sequenced genome [9]. However, less is known regarding library insert size composition.

In this study, we analysed the interplay of most of these factors to evaluate possibilities for further optimization of sequencing projects. Modified versions of the widely used Illumina TruSeq^®^ DNA PCR-free LPP, which enable the creation of libraries with varying library insert sizes, are presented. Furthermore, the relationships between genomic GC content, library insert size and sequencing quality are investigated. Finally, the influence of insert size, read length and sequencing depth on assembly performance of four state of the art assemblers was examined.

## Methods

### Bacterial strains

Table 1 summarizes all bacterial strains used in this study together with the NCBI accession numbers of their already completely assembled reference genome sequences. In addition, strain abbreviations and genomic GC contents are listed.

**Table 1:** Bacterial strains

Bacterial strain	Abbreviation	Reference genome sequences	% GC
*Bacillus cereus* F837/76	Bce	NC_016779.1, NC_016794.1, NC_016780.1	35.4
*Enterococcus faecalis* OG1RF	Efa	NC_017316.1	37.8
*Salmonella* Typhimurium 14028S	Sen	NC_016856.1, NC_016855.1	52.2
*Pseudomonas stutzeri* ATCC 17588	Pst	NC_015740.1	63.9
*Micrococcus luteus* NCTC 2665	Mlu	NC_012803.1	73.0

Sequenced bacterial strains, strain abbreviations, genomic GC-content and corresponding NCBI reference genome sequences

### DNA extraction and quantification

The hexadecyltrimethylammonium bromide (CTAB) method was used to extract genomic DNA. Prior to CTAB DNA extraction bacterial cells were disrupted mechanically with a MP FastPrep^®^-24 instrument (MP Biomedicals, Santa Ana, US-CA) (2 × 45 s at 6.5 m/sec) and 0.1 mm silica beads. Genomic DNA was quantified with a Qubit^®^ 2.0 fluorometer using the Qubit^®^ dsDNA HS assay (Life Technologies, Carlsbad, US-CA).

### PCR-free library preparations

Library preparation was performed following the original Illumina TruSeq^®^ DNA PCR-free LPP (revision A, January 2013, low sample with 550 bps insert size) or a modified version of it (see below). Sequencing libraries resulting from modified LPPs are denoted by IS1-4. IS1 refers to libraries with shorter average insert sizes than IS2 libraries, etc. In contrast, libraries prepared with the original Illumina protocol are denoted by TS. Table 2 gives an overview of the different sequencing library categories.

**Table 2:** Sequencing library categories

Library category	Fragmentation parameters	RB:BB ratios	Avg. insert size (raw data) (bps)	Sequenced genomes
TS	DF 5 %, PIP 175 W, C/B 200, Du 25 s	TruSeq^®^ DNA PCR-free LPP (LS protocol, 550 bps)	641 ± 28	Bce, Efa, Pst, Mlu
IS1	DF 10 %, PIP 175 W, C/B 200, Du 25 s	2.0:1 + 3.8:2	686 ± 33	Bce, Efa, Sen, Pst, Mlu
IS2	DF 2 %, PIP 175 W, C/B 200, Du 30 s	2.2:1 + 4.2:2	990 ± 79	Efa, Sen, Mlu_50_
IS3	DF 2 %, PIP 175 W, C/B 200, Du 20 s	2.3:1 + 4.4:2	1211 ± 78	Bce, Pst, Mlu, Mlu_50_
IS4	DF 2 %, PIP 175 W, C/B 200, Du 10 s	2.4:1 + 4.6:2	1297	Mlu_50_

Settings for genomic DNA fragmentation and fragment size selection during library preparation. TS, original Illumina TruSeq^®^ DNA PCR-free LLP (no modification)

IS1-IS4, categories for library average insert size, where IS1 < IS2 < IS3 < IS4

The term Mlu_50_ refers to the corresponding genome sequenced at 2 × 25 bps. Genomes listed without index were sequenced at 2 × 200 bps throughout

*Bce* *B. cereus,* *Efa* *E. faecalis,* *Pst* *P. stutzeri,* *Mlu* *M. luteu,* *Sen* *S. enterica, RB* Resuspension Buffer, *BB* Bead Buffer, *DF* Duty Factor, *PIP* Peak Incident Power, *C/B* Cycles per Burst, *Du* Duration

### Modified PCR-free library preparation

Modifications to the original LPP were introduced at the following steps: DNA fragmentation, fragment size selection and final library quantification. The different shearing settings and ratios of resuspension buffer (RB) to bead buffer (BB) used during first and second size selection steps applied to generate different libraries are listed in Table 2. The RB:BB ratios listed enabled the capture of DNA fragments within a desired range of length for library categories IS1-4.

In contrast to the original LPP (category TS), each modified LPP (categories IS1-4) starts DNA fragmentation with 52.5 µl of DNA (dissolved in RB) at a concentration of 75 ng/µl instead of 40 ng/µl. Fragmentation was performed using Covaris microTUBES and the S220 focused-ultrasonication system (Covaris, Woburn, US-MA) in frequency sweeping mode at a temperature of 6 °C.

Category IS3 and IS4 libraries had to be prepared twice for one sequencing run due to the high amount of final library concentration necessary to achieve appropriate flow cell cluster densities. Regardless of a library’s category, buffers and reagents were taken from the TruSeq^®^ DNA PCR-free Sample Prep LT kit (Illumina, Inc., San Diego, US-CA) throughout the whole process of library preparation.

### First fragment size selection

In this section, only the preparation of category IS1 sequencing libraries is described in detail. For libraries of categories IS2-4 the detailed RB and BB volumes (and corresponding ratios) applied during first size selection are given in Additional file 1: Table S1.

30 µl of RB (Illumina) was added to 50 µl of fragmented DNA. Subsequently, 40 µl of Agencourt AMPure^®^ XP magnetic bead reagent (Beckman Coulter, Brea, US-CA) was added. The solution was mixed carefully by repeated pipetting and incubated for 5 min. At this step the RB:BB ratio is 80:40 (≙2:1). With this ratio, DNA fragments too large for this category will bind to the magnetic beads. After magnet-induced pelleting of beads for 3 min, 117 µl of the clear supernatant was transferred. Then 70.2 µl RB were added to the supernatant together with 39 µl of 2X magnetic beads. In this case, 2X corresponds to twice the concentration of magnetic beads per volume of BB. Using a higher bead particle concentration enables capturing more DNA fragments of the desired length later on. Again the solution was mixed carefully by repeated pipetting. At this step the RB:BB ratio is 148:78 (≙3.8:2.0). With this ratio, DNA fragments too short for this category will remain in the supernatant, while DNA fragments within the desired range of length will bind to the magnetic beads. After 5 min of incubation and magnet-induced pelleting for 3 min, the clear supernatant was discarded. The remaining bead pellet was washed twice with 200 µl of freshly prepared 80 % ethanol without resuspending while keeping the tube on the magnetic stand. After the second ethanol wash the bead pellet was air-dried for 5 min. Finally, fragmented DNA was eluted from magnetic beads in 52 µl RB.

### End-repair

The end-repair reaction (75 µl) contained 50 µl size selected DNA, 5 µl end repair control (Illumina) and 20 µl end repair mix 2 (Illumina). The enzymatic reaction was carried out on a preheated QBT 2 heat block (Grant Instruments, Cambridge, UK) for 35 min at 30 °C.

### Second fragment size selection

In general, second size selection was performed as the first one using the RB and BB volumes (and corresponding ratios) given in Additional file 1: Table S2. After size selection, the DNA was eluted with 19 µl of RB from the air-dried bead pellet.

### 3′ adenylation and adapter ligation

The adenylation reaction (22.5 µl) contained 15 µl size selected DNA, 1.25 µl A-tailing Control (Illumina) and 6.25 µl A-tailing mix (Illumina) and was carried out on a preheated heat block for 35 min at 37 °C, followed by enzyme inactivation for 5 min at 70 °C. Finally, the reaction was kept on ice for 5 min.

The adapter ligation reaction (26.25 µl) contained 22.5 µl adenylation reaction, 1.25 µl ligation control (Illumina), 1.25 µl ligation mix 2 (Illumina) and 1.25 µl of DNA adapter solution (Illumina) and was carried out on a preheated QBT 2 heat block for 12 min at 30 °C. Finally, enzyme inactivation was achieved by adding 2.5 µl stop ligation buffer (Illumina).

### Final library clean up

25 µl of adapter ligation reaction were purified twice. Each time 25 µl of Agencourt AMPure^®^ XP magnetic bead reagent was added to the reaction (≙RB:BB ratio of 1:1). The reaction was mixed carefully by repeated pipetting. After 5 min of incubation, the beads were pelleted (magnet-induced) for 3 min. Subsequently, the clear supernatant was discarded. Then, the pellet was washed twice with 200 µl of freshly prepared 80 % ethanol without resuspending while keeping the tube on the magnetic stand. After the second ethanol wash, the bead pellet was air-dried for 5 min. After the first purification, DNA was eluted from beads in 25 µl of RB. After the second purification, DNA was eluted in 15 µl RB.

### Library validation and quantification

After second size selection, but before adapter ligation, DNA concentration was measured with a Qubit^®^ 2.0 fluorometer (Qubit^®^ dsDNA HS assay) at ~21 °C room temperature using 1 µl of DNA. Afterwards, 2 µl of DNA were diluted to a final concentration of ~0.3 ng/µl. The average library insert size in bps was verified in triplicate, running 1 µl of diluted DNA using an Agilent high sensitivity DNA chip and a 2100 Bioanalyzer instrument (Agilent Technologies, Santa Clara, US-CA) according to the manufacturer’s instructions. The average of the three independently verified library average insert sizes was defined as the Bioanalyzer-inferred average insert size (*AIS*_*Bio*_).

After adapter ligation and final clean up, DNA concentration was measured (Qubit^®^ dsDNA HS assay) in triplicate using 1 µl of the final sequencing library each time. The average of the three independent measurements was defined as the library’s DNA concentration (*conc*).

The molar concentration of the first final sequencing libraries was estimated by taking the Bioanalyzer-inferred average insert size in bps (*AIS*_*Bio*_) of each library as its actual average insert size in bps (*AIS*). Then, according to [19], Eq. 1 was used to calculate the molarity of each final library (*nM*) dependent on the library’s DNA concentration in ng/µl (*conc*) and actual average insert size in bps (*AIS*).

1 $$nM = \frac{conc}{{\left( {AIS + 120} \right) * 650 \frac{\text{g}}{\text{mol * bps}} }}*10^{6}$$

Subsequently, the molarity of each final library (*nM*) was multiplied by a factor of 1.3 prior to diluting to pools of libraries, since it has been realized from previous sequencing runs and read remapping analysis that the Bioanalyzer system is misestimating insert size distributions. Thus, usage of the multiplication factor 1.3 was a rough adjustment to account for that observation.

Later on it turned out that misestimation inherent to the Bioanalyzer system can be modelled by linear regression to estimate the library average insert sizes more precisely. The final linear regression model is shown in Eq. 2. Its use in combination with Eq. 1 is recommended and should yield most accurate results.

2 $$AIS = 0.564*AIS_{Bio } + 258$$

### Library pooling and loading

Prior to sequencing, final libraries were diluted to 1 nM. Diluted libraries were pooled and an equal volume (ratio 1:1) of freshly prepared 0.1 N NaOH was added. The pool was mixed carefully by repeated pipetting followed by incubation for 5 min on a preheated QBT 2 heat block at 98 °C. After incubation the pool was placed on ice immediately and an equal volume of freshly prepared and pre-chilled 0.1 N HCl (ratio 1:1:1) was added. Again, the chilled pool was mixed carefully by repeated pipetting. Finally, the pool was diluted with pre-chilled hybridization buffer (Illumina) to its desired molarity (pMol) for sequencing.

IS1 libraries were sequenced at 30–35 pMol. IS2 libraries were sequenced at 60–80 pMol and IS3 libraries at 150 pMol. Molarity was determined according to Eq. 1. Quantitative PCR was not performed. Thus, the loaded DNA (pMol) corresponds to total DNA amounts and not to the amount of DNA fragments being double-ligated.

### Genome sequencing and demultiplexing

All libraries sequenced are summarized in Table 2. Sequencing was carried out using the MiSeq sequencing platform (Illumina, Inc., San Diego, US-CA). Libraries were sequenced either with v2 (500-cycle) or v3 (600-cycle) MiSeq Reagent Kits at 2 × 200 bps. In addition, libraries Mlu_50__IS2, Mlu_50__IS3 and Mlu_50__IS4 were sequenced at 2 × 25 bps using a v2 (50-cycle) MiSeq Reagent Kit. After sequencing, read data of pooled libraries was demultiplexed using the on-board MiSeq Reporter software (v2.3.32) of the sequencing platform.

### Trimming and filtering of read data

Read trimming and filtering was done using the NGS QC Toolkit (v2.2.3) [20] with automatic detection of FASTQ variant and considering option 2 (multiplexed DNA libraries) for adapter-contaminated read removal. Furthermore, FastQC (v0.10.1) [21] was used for visual confirmation of high quality (trimmed and filtered) read-pairs. Read data originating from 2 × 200 bps sequenced libraries were trimmed 10 nucleotides from 5′ end and 1 nucleotide from 3′ end. Thus, raw data incorporated into further analysis comprised 2 × 189 bps paired reads. Read data originating from libraries sequenced at 2 × 25 bps (Mlu_50__IS2, Mlu_50__IS3, and Mlu_50__IS4) were not trimmed. After read trimming, raw read data was filtered for high quality read-pairs. If not stated otherwise, reads passed the filter if at least 80 % of their nucleotides had a Phred quality score ≥20 (80;20). Reads losing their forward or reverse counterpart during filtering were discarded from further analysis.

### Remapping of read data

Paired-end reads were aligned to their corresponding NCBI reference genomes (Table 1) using Bwa (v0.6.2) [22] with default parameters, except of setting the maximum number of alignments to output for properly and disconcordant read pairs to 1. Subsequent to read alignment, SAM files were cleaned and coordinate-sorted with the corresponding tools CleanSam and SortSam of the Picard toolkit (v1.84) [23], whereby validation stringency was set to lenient in both cases. Library insert distributions were estimated on cleaned and sorted SAM files using the tool CollectInsertSizeMetrics (Picard toolkit) with lenient validation stringency.

### Linear regression analysis

Linear regression analysis and calculation of coefficients of determination (R^2^) were performed using the corresponding in-build functionality of Gnumeric (v1.10.17) [24].

### Evaluation of factors affecting assembly performance

#### Analyzed factors

The effects of the different factors assembler, insert size, read length and sequencing depth on sequence assembly performance was examined. Tested assemblers were SPAdes (v2.5.1) [25], ABySS (v1.3.3) [26], Velvet (v1.2.10) [27] and Edena (v3.131028) [28]. Tested library categories each having a different insert size were TS (for Bce, Efa, Pst), IS1 (for Bce, Efa, Sen, Pst) and IS2 (for Efa, Sen) or IS3 (for Bce, Pst). Tested read lengths in nucleotides were 100, 125, 150, 175 and 189. Adjustment of read length was done via repeated 3′ read trimming of the unfiltered libraries containing 2 × 189 bps paired reads. Sequencing depths tested were 45 (Bce, Efa, Sen, Pst) and 90 (Bce, Efa, Pst). Sequencing depth (coverage) is defined as $\frac{2LN }{G}$, where *L* is the read length in bps, *N* is the number of read pairs and *G* is the reference genome size in bps. Libraries containing different sequencing depths were created via random read pair drawing from respective trimmed libraries after quality filtering. Filtering was always performed with quality threshold 80;20.

#### Creation of sub-library datasets

From each library sequenced at 2 × 200 bps (Table 2), several sub-libraries were compiled comprising different read lengths and sequencing depths as described above. For each specific read length and sequencing depth 3 read data sets were compiled independently from each other by random read pair drawing, resulting in a total of 300 sub-libraries composed of 100 triplicates for the genomes Bce, Efa, Sen and Pst. Libraries sequenced for Mlu were not part of sub-library creation. For genomes Bce, Efa and Pst the number of created sub-libraries is $3*5*2*3 = 90$ each, since 3 library categories, 5 read lengths and 2 sequencing depths were considered in triplicate for each of these genomes. For Sen the number of created sub-libraries is $2*5*1*3 = 30$, since only two different library categories (IS1, IS2) were prepared for this genome and only one sequencing depth (45) was investigated.

#### Sequence assembly

Each of the 300 sub-libraries were assembled using four different assemblers: SPAdes (v2.5.1), ABySS (v1.3.3), Velvet (v1.2.10) and Edena (v3.131028). In total, $4 * 300 = 1200$ (optimal) assemblies were performed. If not stated otherwise, all assemblers were run with default parameter settings. In general, assembled contigs less than 500 bps in length were discarded from further analysis. This applies also to the calculation of assembly quality metrics like N50, etc.

SPAdes assemblies were performed using its in-build read error correction functionality of BayesHammer [29] by setting the --careful option. Applied $k$-mer combinations were dependent on the sub-library’s read length. For read lengths 189, 175 and 150 the $k$-mer combination was set to $21,33,55,77,99,127$. For read lengths 125 and 100 the $k$-mer combinations were $21,33,55,77,99$ and $21,33,55,77$, respectively.

For each sub-library multiple ABySS and Velvet assemblies were performed within a range of $k$-mer values using stepwise increments of 2. The lower border of the k-mer range was always set to 21. The upper border of the $k$-mer range was dependent on the sub-library’s read length. For read lengths 189, 175, 150, 125 and 100 the upper $k$-mer border was set to 171, 161, 141, 111 and 91, respectively. For each sub-library only the optimal assembly ($k$-mer) was taken into account for further analysis, while all others were rejected. An optimal assembly has the largest N50 value. If there were multiple assemblies ($k$-mers) having the same largest N50, then the one with the smallest number of contigs was chosen as the optimal one. If multiple assemblies with largest N50 and smallest number of contigs occurred, then the assembly with the largest average contig size was defined as optimal. Velvet was always run with non-default parameter settings ‘-alignments yes’, ‘-exp_cov auto’, ‘-ins length auto’, ‘-scaffolding no’ and ‘-read_trkg yes’.

For each sub-library an Edena’s overlaps graph was computed once with a minimum overlap size (option –M) of 21. 3′ end truncation of reads (option –t) was not performed. On the basis of the pre-computed overlaps graph, multiple Edena assemblies were performed for each sub-library within a range of overlap cutoffs (option –m) using stepwise increments of 2. In general, the minimum overlap cutoff was set to 21 and the maximum search distance for paired-end reads (option –peHorizon) was set to 5000. The maximal overlap cutoff was dependent on the sub-library’s read length. For read lengths 189, 175, 150, 125 and 100 the maximal applied overlap cutoffs were 161, 151, 131, 111 and 91, respectively. For each sub-library only the optimal assembly (defined as for ABySS and Velvet) was considered further.

#### Sequence assembly validation

Frequently, scientists use the N50/NG50 metric to judge their assemblies [30]. N50 denotes the contig (or scaffold) comprising 50 % of the assembly size after sorting the set of assembled contigs from longest to shortest. NG50 is defined accordingly, but the size of a reference genome instead of the assembly size is taken into account [18]. Unfortunately, assembly performance judgements based solely on N50/NG50 values make statements about assembly contiguity rather than assembly accuracy [31]. However, in cases where a reference genome is available, the set of assembled contigs (scaffolds) can be aligned to the reference first and contigs exhibiting misassemblies can be broken down at their misassembled regions prior to calculating NG50 values. This strategy, introduced during GAGE, found its expression in the corrected assembly contiguity metric (denoted corrected NG50 in this publication) [13]. Later, a related metric, called NGA50, was defined within the quality assessment tool QUAST [32].

The 300 sub-libraries, composed of 100 triplicates, were assembled using four different assemblers leading to 1200 (optimal) assemblies in total. The 1200 assemblies were separated in 100 assembly sets per assembler, where each assembly set comprised the three assemblies of a specific sub-library triplicate. Assembly sets (derived from the same assembler) were validated against their corresponding reference genome sequences (Table 1) using QUAST (v2.2) [32]. For each assembly of a set the corrected NG50 and NGA50 value was saved. Then, each set was reduced to one assembly validation entry comprising the averages of the corrected NG50 and NGA50 values of the set. In total, 400 assembly validation entries (100 per assembler) were created, whereby each validation entry was unique with regard to its underlying genome and the combination of manifestations of the different factors: assembler, insert size, read length and sequencing depth. In general, QUAST was run with the --ambiguity-usage option set to one and option --gage.

#### Calculation of relative assembly scores

Commonly, assembly validation entries were grouped according to the manifestations of the factor under consideration. Then, each manifestation’s average corrected NG50 and average NGA50 was calculated. Finally, the relative assembly score *S* of each manifestation was determined for each quality metric (corrected NG50, NGA50). This was done by dividing each manifestation’s quality metric’s average by the maximal observed average of the factor analysed. Thus, *S* is defined within $0 < S \le 1$. The closer *S* is to 1, the higher is the positive effect of the corresponding manifestation on sequence assembly.

For the evaluation of assemblers, the assembly validation entries were examined in a genome-wise manner. For all other factors (insert size, read length and sequencing depth), assembly validation entries were analysed per genome and assembler.

## Results

### Optimization of the library preparation protocol

One of the most critical steps during library preparation is insert size selection after genomic DNA shearing. The largest average insert size of a library prepared with the standard Illumina protocol lies within a range of ~550–650 bps. The maximum sequenceable read length is 2 × 300 bps, in general. Increasing insert size should be advantageous for assembly, as it may bridge short repetitive elements. Therefore, adjusted shearing settings during DNA fragmentation and optimized RB to BB ratios during fragment size selection were applied to create sequencing libraries varying in average insert size (categories IS1-3). IS1 libraries had an average insert size of ~690 bps. Insert sizes of IS2 and IS3 libraries were ~990 and ca 1210 bps on average, respectively (Table 2 and Additional file 1: Table S3). However, it turned out that GC-rich libraries tend to have larger average insert sizes in comparison to AT-rich libraries of the same category. Most notably, this GC-content dependent behaviour on insert size was observed for sequenced IS2 and IS3 libraries (Additional file 2: Figure S1).

**Fig. 1:**
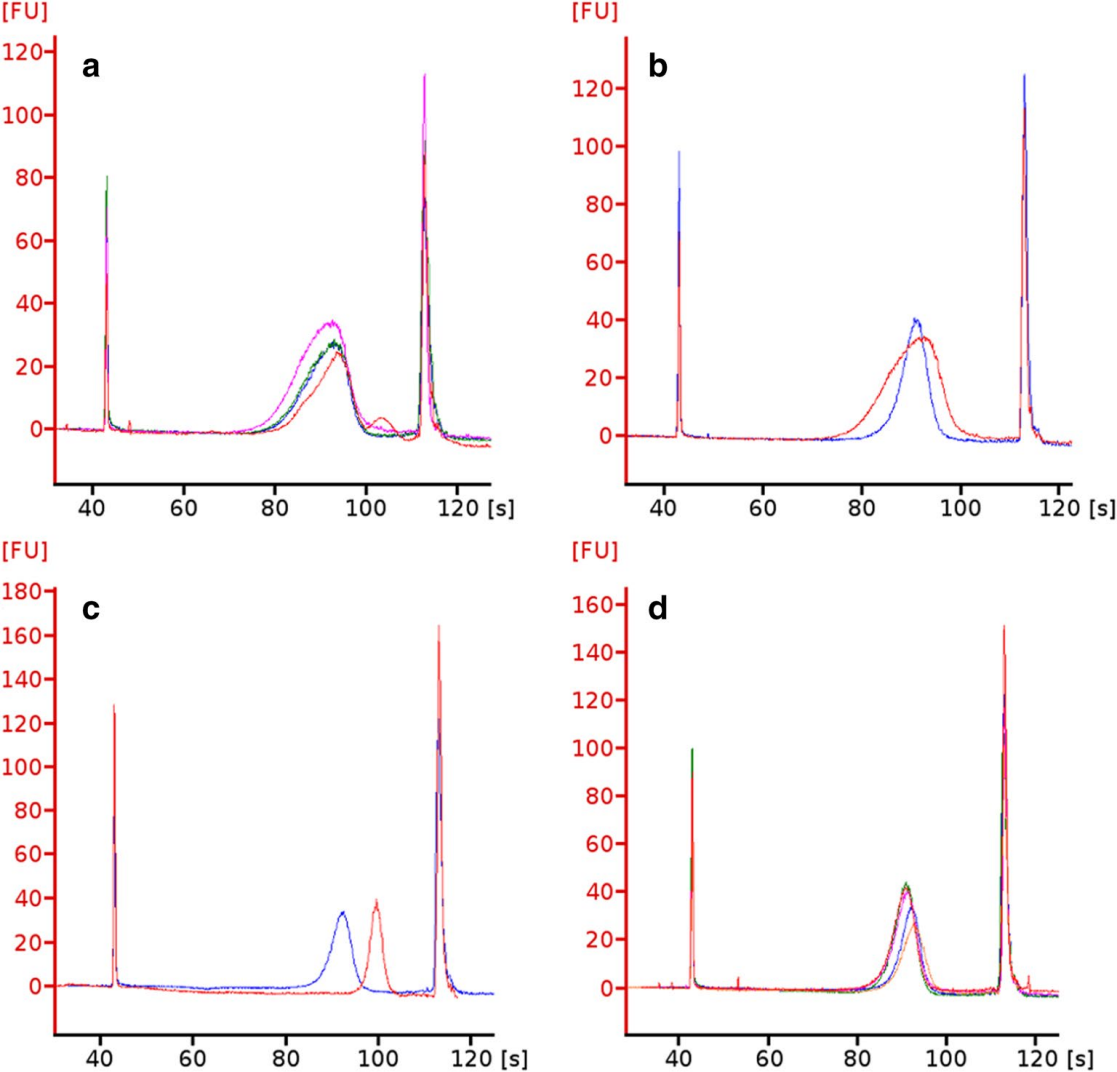
Insert size distributions after second size selection. Data originate from analysis with Bioanalyzer instruments. **a** Insert size distributions of sequencing libraries obtained with the standard Illumina TruSeq^®^ DNA PCR-free LPP have an overall good reproducibility. All sequenced TS libraries are shown. **b** Modifications during DNA fragmentation and insert size selection enabled the creation of sequencing libraries with sharper and more symmetric insert size distributions. Sequencing libraries Efa_TS and Efa_IS1 are illustrated in *red* and *blue*, respectively. **c** In addition, different RB:BB ratios led to sequencing libraries varying in average insert size for the same genome. *Blue*: Pst_IS1, *red*: Pst_IS3. **d** Insert size distribution reproducibility is maintained when using modified LPPs

Figure 1a illustrates the insert size compositions of all libraries prepared with the Illumina TruSeq^®^ DNA PCR-free LPP (category TS). Their shape is asymmetric, i.e. left-tailed, whereas the insert size distributions of the modified protocol are sharper and more symmetric (Fig. 1b). Figure 1c displays libraries of category IS1 and IS3, which differ in average insert size by ca. 540 bps. The very high reproducibility of the modified protocol is demonstrated in Fig. 1d.

**Fig. 2:**
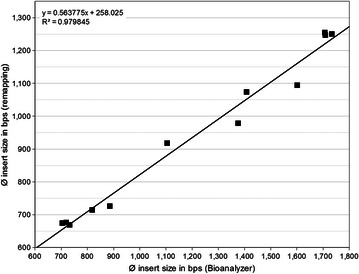
Linear regression of Bioanalyzer deduced and actual average library insert sizes. Calculation of actual library insert sizes was done after remapping of raw read data to respective reference genome sequences. Linear regression analysis revealed a very strong correlation, but the Bioanalyzer system turned out to overestimate library insert sizes

The sharper and (nearly) symmetric library insert size distributions enabled a more precise determination of average insert sizes. However, it turned out that the Bioanalyzer system tends to misestimate library insert sizes. Mapping raw read data from the whole set of sequenced IS1-3 libraries (summarized in Table 2) back to their reference genome sequences (listed in Table 1) revealed a very strong linear correlation (R^2^ ~0.98, *p* value ≪ 0.001) between Bioanalyzer and actual average library insert sizes (Fig. 2). For IS1 libraries average insert sizes were overestimated by the Bioanalyzer by ~22.0 % at maximum. Overestimation increased severely to up to ~46.4 % for IS3 libraries (Additional file 1: Table S3). Since two different Bioanalyzer machines were used independently from each other to determine insert size compositions both showing the same effect, the deviation seems to be of systematic nature. By incorporating the function derived from linear regression analysis into library validation, the molarity of sequencing libraries can be adjusted more precisely.

An additional benefit of the modified LPPs is the reduced amount of reagents required. Preparation of IS1 and IS2 category libraries consumes only half the reagents of the original Illumina protocol. For IS3 libraries larger amounts of DNA (~150 pMol) are needed to create appropriate cluster densities. This leads to a reagent consumption which is similar to that of the standard LPP.

### Impact of average library insert size and genomic GC content on sequencing quality

To evaluate the impact of genomic GC content and average insert size on sequencing quality, strains with GC contents ranging from 35.4–73.0 % (Table 1) and average library insert sizes between 610 bps and 1250 bps (Table 2 and Additional file 1: Table S4) were used. In total, 14 libraries were sequenced on the MiSeq platform at 2 × 200 bps followed by raw read trimming to 2 × 189 bps. Among them, four were prepared with the standard LPP (category TS), 5 were of category IS1, 2 of category IS2 and 3 of category IS3. Remapping of raw reads against their reference genome revealed that TS libraries had similar average insert sizes as their IS1 counterparts of the same genome (Additional file 1: Table S4).

**Fig. 3:**
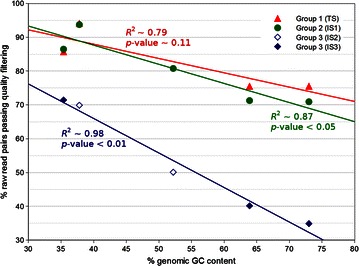
Impact of average library insert size and genomic GC content on sequencing quality. Genomic GC content was plotted against the percentage of raw read pairs passing quality filtering (80;20). Then, libraries were grouped according to their category. Group 1 (*red*) comprises all standard libraries (TS). Group 2 (*green*) covers all libraries of category IS1. Group 3 (*blue*) represents the combined set of IS2 and IS3 libraries

Figure 3 depicts genomic GC contents plotted against the percentage of raw read pairs passing quality filtering for each of the 14 libraries. Regression analysis revealed a general linear decrease in the percentage of raw read pairs passing quality control (QC) as genomic GC content increases. In addition, there was a library specific effect observed, as for libraries with longer insert sizes (group 3), less read pairs are retained after quality filtering regardless of the GC content. Group 1 and 2 showed almost no difference, since libraries of category TS and IS1 have similar average insert sizes.

**Fig. 4:**
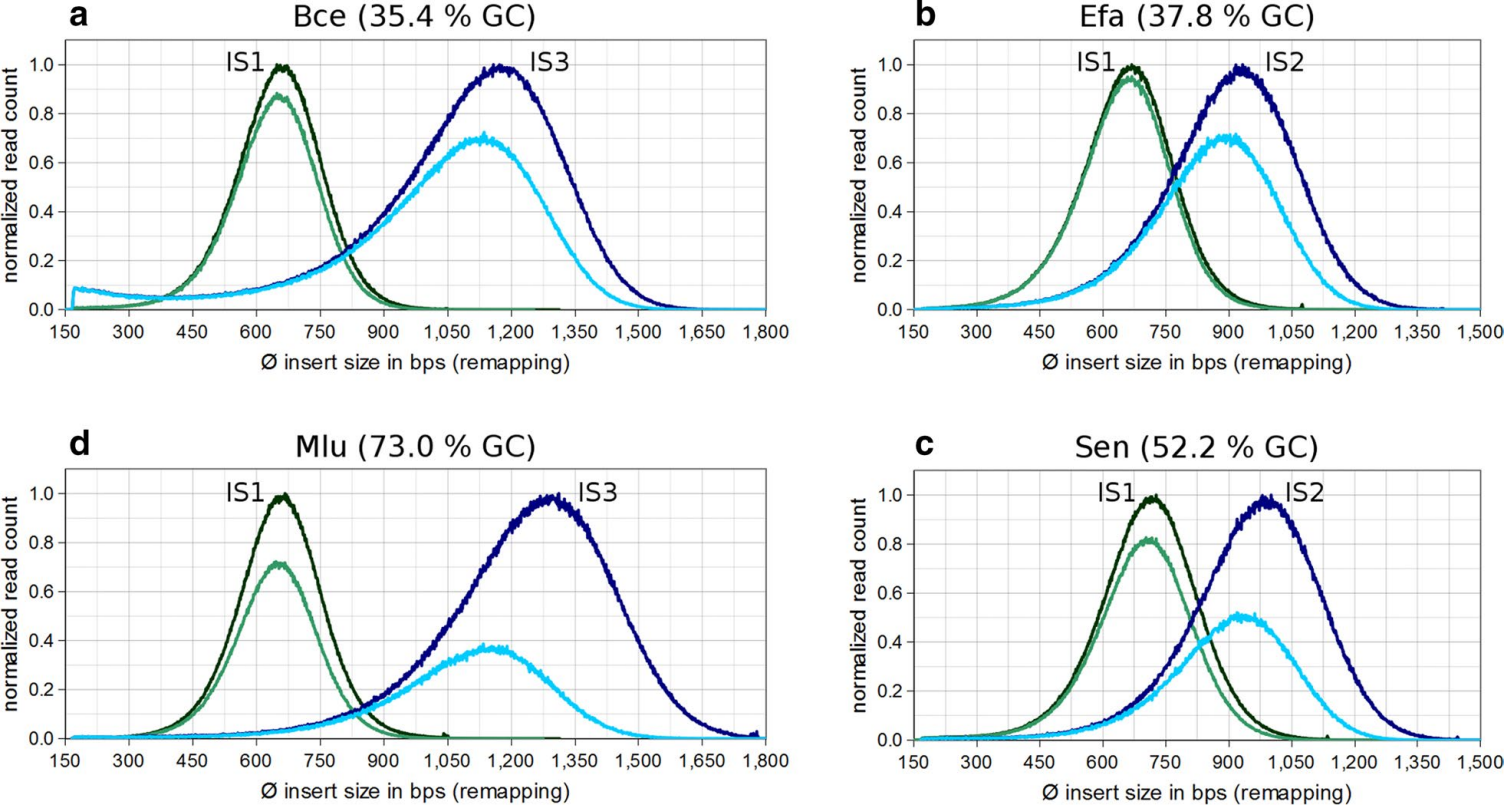
Interplay between insert size, GC content and sequencing quality of a library. Insert size distributions of IS1-3 libraries were plotted prior to and after read quality filtering (80;20). Distributions marked *green* belong to IS1 libraries, whereas distributions marked *blue* are from higher category libraries (IS2 or IS3). *Dark-coloured* distributions are derived from unfiltered libraries. *Light-coloured* distributions are obtained after quality filtering. To make insert size distributions directly comparable to each other, read counts were normalized by the maximal read count (per insert size) of the unfiltered library (of same category and genome). **a** Bce_IS1 and Bce_IS3. **b** Efa_IS1 and Efa_IS2. **c** Sen_IS1 and Sen_IS2. **d** Mlu_IS1 and Mlu_IS3

The loss of reads after quality filtering is accompanied by a reduction in average insert size (Fig. 4). The average library insert size of Bce_IS1 decreases by only ~1.2 % from 669 bps of the raw library (Fig. 4a, dark-green) to 661 bps after quality filtering (Fig. 4a, light-green). Similar observations were made for all other IS1 libraries (Additional file 1: Table S4). Thus, overall read quality of category IS1 libraries is mainly determined by the library’s GC content.

For Efa_IS2 and Sen_IS2, average insert sizes decline by ~4.5 % (Fig. 4b) and ~6.4 % (Fig. 4c) due to filtering. As average insert size and genomic GC content of a library increase further, the effect becomes more pronounced. For instance, the GC rich library Mlu_IS3 (Fig. 4d) performs considerably worse with a reduction of ~11.8 %. The shift of the insert size distribution introduced by quality filtering shows that read pairs originating from longer DNA fragments tend to have worse sequencing quality, since they fail to pass quality filtering disproportionately often. This effect is particularly strong for libraries with high GC content.

**Fig. 5:**
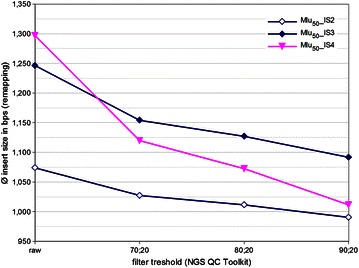
Effect of quality filtering on average library insert size. Average insert sizes for libraries Mlu_50__IS2, Mlu_50__IS3 and Mlu_50__IS4 were plotted as a function of progressive stringency filtering. Most relaxed stringency corresponds to unfiltered libraries (raw). During most discriminative filtering reads are only passing quality control, if at least 90 % of their nucleotides had a Phred quality score ≥20 (90;20)

Thus, the impact of filtering parameters on different insert size distributions was analysed in more detail for a strain with high GC content. The genome of *Micrococcus luteus* NCTC 2665 (73 % GC content) was sequenced at 2 × 25 bps (Mlu_50_) with libraries of category IS2-4. For each category the average insert size of the unfiltered library was estimated via raw read remapping to the reference genome. In addition, each raw library was filtered three times with increasing filter stringency and the average insert sizes of the retained reads were estimated (Fig. 5). The longer the unfiltered insert size distribution (of the raw library), the higher was the reduction in average insert size after quality filtering. Applying the most stringent filter settings (90;20) the library with the smallest inserts (IS2) lost <10 % in average insert size. IS3 lost only little more (~12 %), but the insert size distribution of IS4 was reduced by >20 %.

### Evaluation of factors affecting assembly performance

There is a multitude of factors influencing sequence assembly. Often, such factors are not independent, which makes it a complicated task to infer each factor’s individual impact on sequence assembly. To solve this task a strategy was implemented in which each factor to be benchmarked is rated in the context of other assembly affecting factors. Four different parameters were evaluated: assembler, insert size, read length and sequencing depth. Analyses were performed on genomes of *Bacillus cereus, Enterococcus faecalis, Pseudomonasstutzeri* and *Salmonella enterica*. *Micrococcusluteus* was discarded from this analysis, since remapping of read data revealed that the strain sequenced is very similar, but not identical to the finished reference genome publicly available.

100 sub-library triplicates were created out of the complete dataset as described in “Methods” section. Each member of a triplicate was assembled with SPAdes, ABySS, Velvet and Edena resulting in 100 assembly sets per assembler. Assembly sets were validated against their corresponding reference genomes (Table 1) using QUAST. Then the corrected NG50 and NGA50 values obtained for each assembly set were averaged. By this way 400 assembly validation entries (100 per assembler) were created. Each entry consisted of a pair of average corrected NG50 and NGA50 values.

To nominate the best assembler, assembly validation entries were first grouped according to the respective strain (genome) and then according to the four assemblers. To investigate the influence of insert size, read length and sequencing depth, assembly validations were grouped in a strain and assembler depended manner prior to sub-grouping according to factor manifestations. After sub-grouping, relative assembly scores were calculated as described in “Methods” section. A relative assembly score of 1 is assigned to the best corrected NG50 and NGA50 values. All other manifestations were rated relatively to the best one.

Clearly, SPAdes is the most effective assembler (Table 3). There is only one case in which the NGA50 derived relative assembly score for SPAdes is not 1 (maximal). The impact of average insert size (library category), read length and sequencing depth on assembly performance was analyzed based on SPAdes assemblies (Results obtained for ABySS, Velvet and Edena are summarized in Additional file 1: Tables S6–S14).

Table 4 lists the relative assembly scores for each genome and insert size. There are two obvious tendencies. First, libraries of category IS1 always had better relative assembly scores than libraries of category TS, although the difference varied largely between organisms. In contrast to Pst having a high GC content, the impact of insert size on assembly performance was much more pronounced for the AT-rich genome of Bce or Efa. The second tendency is that maximal relative assembly scores are observed most frequently for larger insert sizes (IS2 or IS3). Both trends deduced from Table 4 illustrate the superiority in SPAdes assembly performance of the modified LPPs (IS1-3) over the standard Illumina protocol (TS).

**Table 3:** Relative assembly scores using different assemblers

Quality metric	Assembler	Genome			
		Bce	Efa	Sen	Pst
Corrected NG50	SPAdes	1	1	1	1
	ABySS	0.69	0.87	0.82	0.70
	Velvet	0.71	0.71	0.77	0.94
	Edena	0.63	0.72	0.63	0.73
	max**	448,776	381,370	292,477	212,702
NGA50	SPAdes	1	1	0.98	1
	ABySS	0.93	0.97	1	0.92
	Velvet	0.58	0.82	0.92	0.96
	Edena	0.40	0.66	0.50	0.66
	max**	733,293	416,896	405,154	238,037

A maximal relative assembly score of 1 refers to the assembler with the best assembly performance. All other assembly scores are expressed as relative fractions of their corresponding maximum. For genomes Bce, Efa and Pst each relative assembly score was calculated on the basis of 30 assembly sets. Assembly sets originated from same-genome sequencing libraries differing in category (average insert size), read length and sequencing depth. For Sen the number of assembly sets per relative assembly score was 10

max** Absolute value in nucleotides of the maximal relative assembly score of the column

**Table 4:** Relative assembly scores for different insert sizes

Quality metric	Insert size	Genome			
		Bce	Efa	Sen	Pst
Corrected NG50	TS	0.54	0.84	n.d.	0.96
	IS1	1	0.95	0.95	0.97
	IS2 or IS3	0.74	1	1	1
	max**	590,346	409,939	300,036	217,864
NGA50	TS	0.53	0.85	n.d.	0.91
	IS1	0.85	0.97	0.96	0.93
	IS2 or IS3	1	1	1	1
	max**	922,992	442,857	405,417	250,853

Relative assembly scores rely on SPAdes assembly validations and are summarized per genome and library category. For Sen each relative assembly score refers to 5 assembly sets. Relative assembly scores of all other genomes comprise 10 assembly sets each

max** Absolute value in nucleotides of the maximal relative assembly score of the column

Table 5 summarizes the relative assembly scores obtained for SPAdes assembled libraries of different read lengths. In most cases, relative assembly scores are maximal (or close to maximal) at read lengths of 175 or 189 bps for both quality metrics. This indicates that stringency (80;20) applied during read quality filtering was sufficient to avoid an impairment of SPAdes assembly performance due to low Phred qualities. Besides, it turned out that shorter read lengths may have drastic negative effects on SPAdes assembly performance. All genomes with low to medium GC-content (Bce, Efa and Sen) obtained their smallest relative assembly scores at the shortest read length of 100 bps.

**Table 5:** Relative assembly scores for different read lengths

Quality metric	Read length	Genome			
		Bce	Efa	Sen	Pst
Corrected NG50	100	0.52	0.68	0.54	0.996
	125	0.82	0.90	0.79	0.995
	150	0.90	0.96	0.81	1
	175	1	1	0.95	0.995
	189	0.91	0.996	1	0.99
	max**	541,348	419,532	358,280	213,754
NGA50	100	0.55	0.95	0.71	1
	125	0.75	1	0.86	0.86
	150	0.78	0.97	0.85	0.96
	175	0.996	0.99	0.94	0.98
	189	1	0.999	1	0.97
	max**	898,406	424,840	455,293	249,244

Read length associated relative assembly scores are listed per genome for SPAdes assemblies. Relative assembly scores for Sen were determined in dependence on 3 assembly sets each. Each other genome comprised 6 assembly sets per relative assembly score

max** Absolute value in nucleotides of the maximal relative assembly score of the column

Finally, the effect of sequencing depth on relative assembly scores was analysed. Regardless of the quality metric examined, maximal relative assembly scores were always reached at a sequencing depth of 90 (Additional file 1: Table S5). However, relative assembly scores grew significantly in a double digit percentage range for Bce only. For Efa and Pst only small enhancements were achieved after doubling sequencing depth from 45 to 90.

## Discussion

For the generation of optimal de novo genome sequences, high quality sequencing data and the choice of optimal data processing tools is essential. When the influence of different parameter settings on sequence and assembly quality is known, this can be taken into account in the setup of a next-generation sequencing experiment.

### Factors affecting assembly performance

#### Assembler

Modern sequence assembly algorithms rest upon one of three different assembly paradigms: greedy, overlap-layout-consensus (OLC) or de Bruijn graph. SPAdes makes use of paired and multi-sized de Bruijn graphs [25]. ABySS and Velvet are classical de Bruijn graph assemblers [26, 27], whereas Edena constitutes an OLC assembler utilizing transitively reduced overlap graphs [28].

Since SPAdes was one of the assemblers performing best during GAGE-B [11], it was not surprising that the assembler also won the small-scale competition of this study (Table 3). Within the set of assemblers investigated, SPAdes represents the most sophisticated tool, which incorporates functionalities for read error correction and contig mismatch correction prior to and after assembly.

#### Insert size

To our knowledge, this is the first study that provides details about the effects of library insert size on assembly performance of bacterial genomes using only a single Illumina paired-end library for sequencing. The effects observed were dependent on the assembler used, but best assemblies were obtained with SPAdes and ABySS using longer insert sizes (IS2 and IS3). In the past, combinations of small and large insert Illumina libraries have been successfully applied. Combining a 100-fold paired-end library with a fivefold mate-pair library, the *Pseudomonas syringae* pv. phaseolicola 1448A genome was assembled in enhanced quality [10]. The assembler ALLPATHS-LG was specifically designed to make use of Illumina paired-end and mate-pair libraries to generate high-quality draft assemblies [33]. During GAGE competition it demonstrated consistently strong performance [13]. However, such approaches require at least two Illumina libraries with mate-pair libraries being difficult to prepare [34]. As an alternative, an increased insert size composition of paired-end libraries can yield benefits in assembly performance. But, the choice of the assembly tool determines whether and to which extent the modified LPPs presented here will lead to enhanced assembly performance.

Indeed, the list of available assemblers is long with each having its own benefits and disadvantages. Unfortunately, validating them all would be far beyond the scope of this work. The sequencing libraries provided in the course of this study should form an excellent test set to validate each assembler’s performance in the context of library insert size. In addition, it may help computational biologists to improve the gap filling and repeat bridging capacities of their already existing tools. Raw sequencing read data can be downloaded from the sequencing read archive (see “Availability of supporting data” section).

#### Read length

**Fig. 6:**
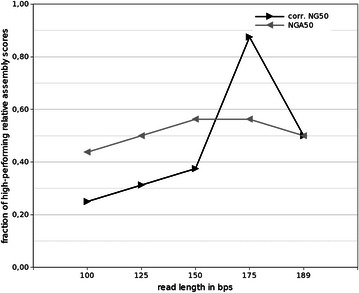
Influence of read length on assembly performance. Shown is the fraction of top-performing assemblies as a function of library read length. Assemblies are defined to be top-performing, if their relative assembly scores are greater or equal to 0.95. Fractions were calculated comprising all investigated genomes and assemblers (Table 5; Additional file 1: Tables S7, S10 and S13)

In contrast to insert size, the impact of a library’s read length on assembly performance seems to follow more general principles. The fraction of very high relative assembly scores in the range of [0.95, 1] was determined over all investigated genomes and assemblers for each read length (Fig. 6). Regardless of the quality metric investigated, the overall pattern is similar. The shortest read length of 100 bps achieves only few high relative assembly scores. Then, the number of high scores increases and reaches maximal values at 175 bps. At 189 bps there is a decline. This curve progression is for the most part in line with what is expected from theory. Repetitive sequences are causing gaps in sequence assembly as long as read length (or the insert size of a read pair) is not capable of spanning the repeat. As read length increases, the portion of unique sequences elevates too, thereby reducing the amount of sequences which had been previously (at shorter read lengths) repetitive [9]. It seems therefore advisable to increase read length. The maximum read length using the Illumina MiSeq platform now is 2 × 300 bps with insert sizes of up to ~1500 bps, but in most bacteria the longest repetitive element constitutes the 16S rDNA operon with 5–7 kbps [35]. PacBio’s single molecule, real time sequencing technology is capable of spanning the rDNA operon by the generation of continuous long reads. Yet, the use of hybrid error correction or self-correction approaches to enhance PacBio’s continuous long read accuracy resulted in assemblies of unprecedented contiguity [35, 36]. However, PacBio sample preparation and sequencing is more costly compared to Illumina [35].

The reason for the decreasing fraction of top-performing relative assembly scores for a read length of 189 bps observed in this study is not clear. Presumably, this is due to base calling errors accumulating at the 3′ ends of Illumina reads, interfering with the assembly process and counteracting the enhanced repeat resolution capacity. It has been shown that read error correction prior to sequence assembly can substantially improve assembly performance [37, 38]. This hypothesis is underlined by the fact that in contrast to the average values of Fig. 6, maximum relative assembly scores for SPAdes were mostly achieved at a read length of 189 bps (Table 5). Since solely SPAdes made use of read error correction prior to sequence assembly, its improved assembly performance capacity at a read length of 189 bps may be the consequence of diminished base calling error rates at 3′ read ends.

#### Sequencing depth

Several studies already addressed the question of an optimal sequencing depth for de novo bacterial genome assembly [10, 14, 16]. Assembly of simulated Illumina read data at different sequencing depths demonstrated the existence of DCAPs between 20- and 40-fold. An assembler’s DCAP was defined as the depth of coverage at which a N50 plateau is reached [16]. Increasing sequencing coverage beyond DCAP does not result in a significant gain of the N50 value anymore. Although sequencing of small genomes is easily realizable at very high sequencing depths (>100-fold) with current Illumina sequencing platforms, its efficiency remains questionable, as it is accompanied by higher costs (occupied portion of flow cell per sequenced genome) and computation effort (runtime, RAM usage). Investigations on the depth of coverage for SPAdes assemblies of larger insert size libraries with read lengths of 189 bps (Additional file 2: Figure S2, S3) revealed a saturation of corrected NG50 and NGA50 values between 30- and 80-fold coverage. This is in line with observations described in the literature. *P. syringae* pv. phaseolicola 1448A genome assemblies plateaued at a sequencing depth of 100-fold [10]. The optimal sequencing depth to assemble the *Escherichia coli* MG1655 genome was around 50-fold for a great majority of assemblers [14]. From our experience, sequencing depths in the range from 50- to 80-fold should give a good compromise between the costs of sequencing and reaching DCAP for the majority of bacterial genomes and assemblers.

#### Eukaryotic genome sequencing

Eukaryotic genomes harbour more complex and longer repeat structures than can be found in prokaryotes. Using a single Illumina library will not be sufficient to generate adequate genome assemblies. However, using a mixture of Illumina libraries with different insert sizes is more effective. Libraries with shorter insert sizes will be capable to resolve smaller repeats, while the ones with large inserts operate on longer repetitive elements [9]. The genome of *Ailuropoda melanoleura* (giant panda) was sequenced using 37 Illumina libraries with insert sizes of 150, 500 bps, 2, 5 and 10 kbps [39]. The modified LPPs presented in this study will increase the repertoire of preparable insert size distributions in such Illumina-only sequencing projects. However, emerged hybrid approaches combining different sequencing technologies may be more promising in eukaryotic sequencing projects [36, 40, 41].

### Optimized library preparation

Although it turned out that longer insert sizes are advantageous for assembly of bacterial genomes, it needs to be considered that they are detrimental for read quality and lead to a substantial loss of reads. As this gradually increases with rising GC content, it would be reasonable to choose the library preparation parameters according to the GC content of DNA. Accordingly, libraries with longer insert sizes (IS2-3) are only recommended for AT rich genomes. However, an increased coverage compensates the loss of reads during quality filtering. Then, sufficient reads may also be obtained for GC rich genomes, but to a higher price, as fewer genomes can be sequenced in parallel during the same run. If enlarged average library insert sizes are desired for sequencing GC rich genomes, a shorter read length could be chosen. As sequencing quality of Illumina reads decreases steadily from 5′ to 3′ end (Additional file 2: Figures S4, S5), shorter read lengths will enable a greater portion of read pairs with large inserts to pass quality filtering.

## Conclusions

In this study modified versions of the widely used Illumina TruSeq^®^ DNA PCR-free library preparation protocol were presented that enable the generation of sequencing libraries with longer average insert sizes. This leads to substantial assembly improvements using SPAdes, which is currently one of the best performing assemblers for bacterial de novo genome assembly. Through the introduced modifications fewer reagents are consumed.

For sequencing Illumina paired-end libraries, maximizing read length and insert size appears reasonable, since both characteristics can elevate the repeat bridging and resolution capacity of current state of the art assemblers. Unfortunately, read length and insert size are negatively correlated with sequencing quality, which is particularly pronounced for high GC content genomes. Major improvements could be achieved if these negative correlations still inherent to the sequencing process were diminished.

## Additional files

10.1186/s13104-016-2072-9 Applied RB:BB ratios during first size selection. **Table S2.** Applied RB:BB ratios during second size selection. **Table S3.** Average insert sizes of sequenced libraries. **Table S4.** Average library insert sizes before and after read quality filtering. **Table S5.** Relative assembly scores for factor sequencing depth (SPAdes assemblies). **Table S6.** Relative assembly scores for factor library category (ABySS assemblies). **Table S7.** Relative assembly scores for factor read length (ABySS assemblies). **Table S8.** Relative assembly scores for factor sequencing depth (ABySS assemblies). **Table S9.** Relative assembly scores for factor library category (Velvet assemblies). **Table S10.** Relative assembly scores for factor read length (Velvet assemblies). **Table S11.** Relative assembly scores for factor sequencing depth (Velvet assemblies). **Table S12.** Relative assembly scores for factor library category (Edena assemblies). **Table S13.** Relative assembly scores for factor read length (Edena assemblies). **Table S14.** Relative assembly scores of factor sequencing depth (Edena assemblies). 
10.1186/s13104-016-2072-9 Intra-category GC-content dependent characteristics of average insert size. **Figure S2.** Corrected NG50 plateau analysis of libraries with a read length of 189 nucleotides. **Figure S3.** NGA50 plateau analysis of libraries with a read length of 189 nucleotides. **Figure S4.** Boxplot representation of Phred scores. **Figure S5.** FASTQC boxplot representation of Phred scores.

## Abbreviations

BB: bead buffer
Bce: *Bacillus cereus* F837/76
CTAB: hexadecyltrimethylammonium bromide
C/B: cycles per burst
DF: duty factor
Du: duration
Efa: *Enterococcus faecalis* OG1RF
GAGE-B: genome assembly gold-standard evaluation for bacteria
IS1: library of category ‘Insert Size 1’
IS2: library of category ‘Insert Size 2’
IS3: library of category ‘Insert Size 3’
IS4: library of category ‘Insert Size 4’
LPP: library preparation procedure
Mlu: *Micrococcus luteus* NCTC 2665
NCBI: National center for biotechnology information
NGS: next-generation sequencing
OLC: overlap-layout-consensus
PIP: peak incident power
Pst: *Pseudomonas stutzeri* ATCC 17588
QC: quality control
RB: resuspension buffer
Sen: *Salmonella* typhimurium 14028S
SRA: sequence read archive
TS: library of category ‘TruSeq^®^ DNA PCR-free’

## References

[1] Mardis ER (2008). The impact of next-generation sequencing technology on genetics. Trends Genet.

[2] Bentley DR, Balasubramanian S, Swerdlow HP, Smith GP, Milton J, Brown CG, Hall KP, Evers DJ, Barnes CL, Bignell HR (2008). Accurate whole human genome sequencing using reversible terminator chemistry. Nature.

[3] Global NGS market: most lucrative sector of the genomics industry. 2013. http://www.companiesandmarkets.com/Market/Healthcare-and-Medical/Market-Research/Next-Generation-Sequencing-NGS-Market-Global-Forecast-to-2017/RPT1167432. Accessed 28 Apr 2016.

[4] Aird D, Ross MG, Chen WS, Danielsson M, Fennell T, Russ C, Jaffe DB, Nusbaum C, Gnirke A (2011). Analyzing and minimizing PCR amplification bias in Illumina sequencing libraries. Genome Biol.

[5] Kozarewa I, Ning Z, Quail MA, Sanders MJ, Berriman M, Turner DJ (2009). Amplification-free Illumina sequencing-library preparation facilitates improved mapping and assembly of (G + C)-biased genomes. Nat Methods.

[6] Oyola SO, Otto TD, Gu Y, Maslen G, Manske M, Campino S, Turner DJ, Macinnis B, Kwiatkowski DP, Swerdlow HP (2012). Optimizing Illumina next-generation sequencing library preparation for extremely AT-biased genomes. BMC Genom.

[7] Quail MA, Kozarewa I, Smith F, Scally A, Stephens PJ, Durbin R, Swerdlow H, Turner DJ (2008). A large genome center’s improvements to the Illumina sequencing system. Nat Methods.

[8] Tyler AD, Christianson S, Knox NC, Mabon P, Wolfe J, Van Domselaar G, Graham MR, Sharma MK (2016). Comparison of sample preparation methods used for the next-generation sequencing of *Mycobacterium tuberculosis*. PLoS One.

[9] Schatz MC, Delcher AL, Salzberg SL (2010). Assembly of large genomes using second-generation sequencing. Genome Res.

[10] O’Brien HE, Gong Y, Fung P, Wang PW, Guttman DS (2011). Use of low-coverage, large-insert, short-read data for rapid and accurate generation of enhanced-quality draft Pseudomonas genome sequences. PLoS One.

[11] Magoc T, Pabinger S, Canzar S, Liu X, Su Q, Puiu D, Tallon LJ, Salzberg SL (2013). GAGE-B: an evaluation of genome assemblers for bacterial organisms. Bioinformatics.

[12] Lee H, Tang H (2012). Next-generation sequencing technologies and fragment assembly algorithms. Methods Mol Biol.

[13] Salzberg SL, Phillippy AM, Zimin A, Puiu D, Magoc T, Koren S, Treangen TJ, Schatz MC, Delcher AL, Roberts M (2012). GAGE: a critical evaluation of genome assemblies and assembly algorithms. Genome Res.

[14] Desai A, Marwah VS, Yadav A, Jha V, Dhaygude K, Bangar U, Kulkarni V, Jere A (2013). Identification of optimum sequencing depth especially for de novo genome assembly of small genomes using next generation sequencing data. PLoS One.

[15] Haridas S, Breuill C, Bohlmann J, Hsiang T (2011). A biologist’s guide to de novo genome assembly using next-generation sequence data: a test with fungal genomes. J Microbiol Methods.

[16] Lin Y, Li J, Shen H, Zhang L, Papasian CJ, Deng HW (2011). Comparative studies of de novo assembly tools for next-generation sequencing technologies. Bioinformatics.

[17] Bradnam KR, Fass JN, Alexandrov A, Baranay P, Bechner M, Birol I, Boisvert S, Chapman JA, Chapuis G, Chikhi R (2013). Assemblathon 2: evaluating de novo methods of genome assembly in three vertebrate species. Gigascience.

[18] Earl D, Bradnam K, St John J, Darling A, Lin D, Fass J, Yu HO, Buffalo V, Zerbino DR, Diekhans M (2011). Assemblathon 1: a competitive assessment of de novo short read assembly methods. Genome Res.

[19] UCI genomics high throughput facility. 2010. http://ghtf.biochem.uci.edu/content/illumina-guidelines. Accessed 28 Apr 2016.

[20] Patel RK, Jain M (2012). NGS QC toolkit: a toolkit for quality control of next generation sequencing data. PLoS One.

[21] FastQC—a quality control tool for high throughput sequence data. 2010. http://www.bioinformatics.babraham.ac.uk/projects/fastqc/. Accessed 28 Apr 2016.

[22] Li H, Durbin R (2010). Fast and accurate long-read alignment with Burrows-Wheeler transform. Bioinformatics.

[23] Picard command-line tools. http://broadinstitute.github.io/picard/. Accessed 28 Apr 2016.

[24] Gnumeric Spreadsheet. 2004. http://www.gnumeric.org/. Accessed 28 Apr 2016.

[25] Bankevich A, Nurk S, Antipov D, Gurevich AA, Dvorkin M, Kulikov AS, Lesin VM, Nikolenko SI, Pham S, Prjibelski AD (2012). SPAdes: a new genome assembly algorithm and its applications to single-cell sequencing. J Comput Biol.

[26] Simpson JT, Wong K, Jackman SD, Schein JE, Jones SJ, Birol I (2009). ABySS: a parallel assembler for short read sequence data. Genome Res.

[27] Zerbino DR, Birney E (2008). Velvet: algorithms for de novo short read assembly using de Bruijn graphs. Genome Res.

[28] Hernandez D, Tewhey R, Veyrieras JB, Farinelli L, Osteras M, Francois P, Schrenzel J (2014). De novo finished 2.8 Mbp Staphylococcus aureus genome assembly from 100 bp short and long range paired-end reads. Bioinformatics.

[29] Nikolenko SI, Korobeynikov AI, Alekseyev MA (2013). BayesHammer: Bayesian clustering for error correction in single-cell sequencing. BMC Genom.

[30] Nagarajan N, Pop M (2013). Sequence assembly demystified. Nat Rev Genet.

[31] Paszkiewicz K, Studholme DJ (2010). De novo assembly of short sequence reads. Brief Bioinform.

[32] Gurevich A, Saveliev V, Vyahhi N, Tesler G (2013). QUAST: quality assessment tool for genome assemblies. Bioinformatics.

[33] Gnerre S, Maccallum I, Przybylski D, Ribeiro FJ, Burton JN, Walker BJ, Sharpe T, Hall G, Shea TP, Sykes S (2011). High-quality draft assemblies of mammalian genomes from massively parallel sequence data. Proc Natl Acad Sci U S A.

[34] Peng Z, Zhao Z, Nath N, Froula JL, Clum A, Zhang T, Cheng JF, Copeland AC, Pennacchio LA, Chen F (2012). Generation of long insert pairs using a Cre-LoxP Inverse PCR approach. PLoS One.

[35] Koren S, Harhay GP, Smith TP, Bono JL, Harhay DM, McVey SD, Radune D, Bergman NH, Phillippy AM (2013). Reducing assembly complexity of microbial genomes with single-molecule sequencing. Genome Biol.

[36] Koren S, Schatz MC, Walenz BP, Martin J, Howard JT, Ganapathy G, Wang Z, Rasko DA, McCombie WR, Jarvis ED (2012). Hybrid error correction and de novo assembly of single-molecule sequencing reads. Nat Biotechnol.

[37] Kelley DR, Schatz MC, Salzberg SL (2010). Quake: quality-aware detection and correction of sequencing errors. Genome Biol.

[38] Schroder J, Schroder H, Puglisi SJ, Sinha R, Schmidt B (2009). SHREC: a short-read error correction method. Bioinformatics.

[39] Li R, Fan W, Tian G, Zhu H, He L, Cai J, Huang Q, Cai Q, Li B, Bai Y (2010). The sequence and de novo assembly of the giant panda genome. Nature.

[40] Huang S, Li R, Zhang Z, Li L, Gu X, Fan W, Lucas WJ, Wang X, Xie B, Ni P (2009). The genome of the cucumber Cucumis sativus L. Nat Genet.

[41] Ikegami T, Inatsugi T, Kojima I, Umemura M, Hagiwara H, Machida M, Asai K (2015). Hybrid de novo genome assembly using MiSeq and SOLiD short read data. PLoS One.

