# Supplementary material for: Optimized Illumina PCR-free library preparation for bacterial whole genome sequencing and analysis of factors influencing de novo assembly
Source: BMC Res Notes. 2016 May 12;9:269. doi: 10.1186/s13104-016-2072-9 (PMC4864918; doi:10.1186/s13104-016-2072-9)
Supplement: Supplementary file 1 — 10.1186/s13104-016-2072-9 Applied RB:BB ratios during first size selection. Table S2. Applied RB:BB ratios during second size selection. Table S3. Average insert sizes of sequenced libraries. Table S4. Average library insert sizes before and after read quality filtering. Table S5. Relative assembly scores for factor sequencing depth (SPAdes assemblies). Table S6. Relative assembly scores for factor library category (ABySS assemblies). Table S7. Relative assembly scores for factor read length (ABySS assemblies). Table S8. Relative assembly scores for factor sequencing depth (ABySS assemblies). Table S9. Relative assembly scores for factor library category (Velvet assemblies). Table S10. Relative assembly scores for factor read length (Velvet assemblies). Table S11. Relative assembly scores for factor sequencing depth (Velvet assemblies). Table S12. Relative assembly scores for factor library category (Edena assemblies). Table S13. Relative assembly scores for factor read length (Edena assemblies). Table S14. Relative assembly scores of factor sequencing depth (Edena assemblies). [file 13104_2016_2072_MOESM1_ESM.pdf]

Additional file 1

Table S1 – Applied RB:BB ratios during first size selection

STEP	ACTION	*INFORMATION*	LIBRARY CATEGORY			
			IS1	IS2	IS3	IS4
1	Take 50 µl fragmented DNA	*DNA is dissolved in RB*	50.0	50.0	50.0	50.0
2	Add RB		+ 30.0	+ 38.0	+ 42.0	+ 46.0
		Volume of RB after step 2	***80.0***	***88.0***	***92.0***	***96.0***
3	Add 1X Beads		+ 40.0	+ 40.0	+ 40.0	+ 40.0
		Total volume (RB and BB) after step 3	***120.0***	***128.0***	***132.0***	***136.0***
		RB:BB ratio by volume	*80.0:40.0 (2.0:1.0)*	*88.0:40.0 (2.2:1.0)*	*92.0:40.0 (2.3:1.0)*	*96.0:40.0 (2.4:1.0)*
4	Take supernatant		117.0	125.0	129.0	133.0
5	Add RB		+ 70.2	*+ 78.3*	*+ 82.1*	*+86.0*
6	Add 2X Beads		+ 39.0	+ 39.1	+ 39.1	+ 39.1
		Total volume (RB and BB) after step 6	***226.2***	***242.4***	***250.2***	***258.1***
		RB:BB ratio by volume	*148.2:78.0 (~ 3.8:2.0)*	*164.2:78.2 (~ 4.2:2.0)*	*172.0:78.2 (~ 4.4:2.0)*	*179.9:78.2 (~ 4.6:2.0)*
7	Take Bead pellet and proceed as described in Materials & Methods		…	…	…	…

Color scheme:

Blue - Volumes in µl of Resuspension Buffer (RB) from the Illumina TruSeq® DNA PCR-free Sample Preparation Kit.

Red - Volumes in µl of Bead Buffer (BB) containing 1X or 2X concentrated magnetic bead particles of the Agencourt AMPure® XP Kit.

Magenta - Volumes in µl of mixtures containing RB and BB.

Gray shaded lines - Information about volumes of mixtures containing RB and BB together with their underlying RB:BB ratios in black parentheses.

Table S2 – Applied RB:BB ratios during second size selection

STEP	ACTION	*INFORMATION*	LIBRARY CATEGORY			
			**IS1**	**IS2**	**IS3**	**IS4**
1	Take 70 µl end-repaired DNA	*DNA is dissolved in RB*	70.0	70.0	70.0	70.0
2	Add RB		+ 10.0	+ 18.0	+ 22.0	+ 26.0
		Volume of RB after step 2	***80.0***	***88.0***	***92.0***	***96.0***
3	Proceed with step 3 of Table S1		…	…	…	…

Color scheme:

Blue - Volumes in µl of Resuspension Buffer (RB) from the Illumina TruSeq® DNA PCR-free Sample Preparation Kit.

Table S3 - Average insert sizes of sequenced libraries

library sequenced*	average insert size** in bps		% overestimation of Bioanalyzer
	Bioanalyzer	raw read remapping***	
Efa_IS1	704	674	4.45
Mlu_IS1	719	675	6.52
Bce_IS1	732	669	9.42
Pst_IS1	819	714	14.71
Sen_IS1	886	726	22.04
Efa_IS2	1,105	918	20.37
Sen_IS2	1,376	978	40.70
Mlu_{50}_IS2	1,408	1,074	31.10
Bce_IS3	1,602	1,094	46.44
Mlu_IS3	1,707	1,255	36.02
Mlu_{50}_IS3	1,709	1,246	37.16
Pst_IS3	1,734	1,250	38.72

* Sequenced bacterial strains and library categories are summarized in Table 1 and 2

** Averages were rounded to nearest integer value

*** Values include 20 nucleotides from 5’ read trimming

Table S4 - Average library insert sizes before and after read quality filtering

library sequenced*	% raw read pairs passing quality filtering	average insert size** in bps		% decrease of average insert size
		raw read remapping	high quality read remapping***	
Bce_TS	85.66	654	632	3.36
Bce_IS1	86.45	669	661	1.20
Bce_IS3	71.43	1,094	1,038	5.12
Efa_TS	94.02	610	597	2.13
Efa_IS1	93.72	674	668	0.89
Efa_IS2	69.81	918	877	4.47
Sen_IS1	80.74	726	713	1.79
Sen_IS2	50.09	978	915	6.44
Pst_TS	75.50	673	647	3.86
Pst_IS1	71.22	714	698	2.24
Pst_IS3	40.10	1,250	1,121	10.32
Mlu_TS	75.42	626	604	3.51
Mlu_IS1	70,89	675	664	1.63
Mlu_IS3	34.81	1,255	1,107	11.79

* Sequenced bacterial strains and library categories are summarized in Table 1 and 2

** Averages were rounded to nearest integer value. Values include 20 nucleotides from 5’ read trimming.

*** Read pairs are defined to be of high quality, if at least 80% of each read’s nucleotides have a Phred quality score ≥ 20.

Table S5 – Relative assembly scores for factor sequencing depth (SPAdes assemblies)

quality metric	sequencing depth	genome* Bce	Efa	Pst
corrected NG50	**45**	0.85	0.94	0.99
	**90**	1	1	1
	max*	*485,517*	*393,034*	*213,539*
NGA50	**45**	0.78	0.96	0.96
	**90**	1	1	1
	max*	*821,716*	*424,647*	*242,739*

* Relative assembly scores were rounded to the second decimal.

** *max* refers to the absolute value in nucleotides for the maximal relative assembly score of the column. *max* values represent averages that were rounded to nearest integer.

Table S6 – Relative assembly scores for factor library category (ABySS assemblies)

quality metric	insert size	genome* Bce	Efa	Sen	Pst
corrected NG50	**TS**	0.81	1	n.d.	0.74
	**IS1**	0.82	0.99	0.96	0.69
	**IS2 or IS3**	1	0.98	1	1
	max*	*353,214*	*335,452*	*246,064*	*183,457*
NGA50	**TS**	0.86	0.85	n.d.	0.98
	**IS1**	0.83	0.87	0.77	0.95
	**IS2 or IS3**	1	1	1	1
	max*	*759,632*	*445,272*	*458,061*	*223,858*

* Relative assembly scores were rounded to the second decimal.

** *max* refers to the absolute value in nucleotides for the maximal relative assembly score of the column. *max* values represent averages that were rounded to nearest integer.

Table S7 – Relative assembly scores for factor read length (ABySS assemblies)

quality metric	read length	genome*			
		Bce	Efa	Sen	Pst
corrected NG50	**100**	0.89	0.78	0.85	1
	**125**	0.94	0.82	0.999	0.80
	**150**	0.72	0.91	0.94	0.75
	**175**	1	0.99	1	0.90
	**189**	0.75	1	0.998	0.86
	max**	*360,330*	*368,221*	*251,266*	*172,209*
NGA50	**100**	0.74	0.96	0.80	0.98
	**125**	0.75	0.96	1	0.99
	**150**	1	0.94	0.98	0.97
	**175**	0.94	1	0.92	0.99
	**189**	0.90	0.96	0.90	1
	max**	*784,943*	*418,320*	*440,832*	*221,474*

* Relative assembly scores were rounded to the second decimal. In cases where a clear distinction between maximal and non-maximal scores would no longer be possible, rounding was extended to the first decimal place capable of being differentiated.

** *max* refers to the absolute value in nucleotides for the maximal relative assembly score of the column. *max* values represent averages that were rounded to nearest integer.

Table S8 – Relative assembly scores for factor sequencing depth (ABySS assemblies)

quality metric	sequencing depth	genome*		
		Bce	Efa	Pst
corrected NG50	**45**	0.74	0.70	0.77
	**90**	1	1	1
	max**	*355,983*	*389,866*	*167,776*
NGA50	**45**	0.76	0.90	0.96
	**90**	1	1	1
	max**	*775,233*	*424,126*	*223,188*

* Relative assembly scores were rounded to the second decimal.

** *max* refers to the absolute value in nucleotides for the maximal relative assembly score of the column. *max* values represent averages that were rounded to nearest integer.

Table S9 – Relative assembly scores for factor library category (Velvet assemblies)

quality metric	insert size	genome* Bce	Efa	Sen	Pst
corrected NG50	**TS**	0.34	1	n.d.	0.94
	**IS1**	1	0.88	1	0.96
	**IS2 or IS3**	0.35	0.82	0.94	1
	max**	*567,563*	*300,545*	*231,909*	*206,284*
NGA50	**TS**	0.30	1	n.d.	0.97
	**IS1**	1	0.85	1	0.97
	**IS2 or IS3**	0.37	0.87	0.92	1
	max**	*757,995*	*328,125*	*390,653*	*232,915*

* Relative assembly scores were rounded to the second decimal.

** *max* refers to the absolute value in nucleotides for the maximal relative assembly score of the column. *max* values represent averages that were rounded to nearest integer.

Table S10 – Relative assembly scores for factor read length (Velvet assemblies)

quality metric	read length	genome* Bce	Efa	Sen	Pst
corrected NG50	**100**	0.66	0.69	0.80	1
	**125**	0.82	0.69	0.83	0.91
	**150**	0.96	0.85	0.83	0.94
	**175**	1	1	1	0.95
	**189**	0.98	0.95	0.86	0.89
	max**	*373,968*	*323,927*	*259,132*	*212,449*
NGA50	**100**	0.78	0.81	1	1
	**125**	0.87	0.87	0.99	0.97
	**150**	1	0.84	0.95	0.96
	**175**	0.89	1	0.81	0.91
	**189**	0.75	0.99	0.84	0.93
	max**	*490,929*	*379,037*	*408,297*	*110,392*

* Relative assembly scores were rounded to the second decimal. In cases where a clear distinction between maximal and non-maximal scores would no longer be possible, rounding was extended to the first decimal place capable of being differentiated.

** *max* refers to the absolute value in nucleotides for the maximal relative assembly score of the column. *max* values represent averages that were rounded to nearest integer.

Table S11 – Relative assembly scores for factor sequencing depth (Velvet assemblies)

quality metric	sequencing depth	genome*		
		Bce	Efa	Pst
corrected NG50	**45**	0.70	0.59	0.87
	**90**	1	1	1
	max**	*375,042*	*341,912*	*213,284*
NGA50	**45**	0.73	0.61	0.90
	**90**	1	1	1
	max**	*487,795*	*425,447*	*240,426*

* Relative assembly scores were rounded to the second decimal.

** *max* refers to the absolute value in nucleotides for the maximal relative assembly score of the column. *max* values represent averages that were rounded to nearest integer.

Table S12 – Relative assembly scores for factor library category (Edena assemblies)

quality metric	insert size	genome*			
		Bce	Efa	Sen	Pst
corrected NG50	**TS**	0.20	1	n.d.	1
	**IS1**	1	0.99	1	0.96
	**IS2 or IS3**	0.33	0.77	0.77	0.75
	max**	*551,178*	*296,040*	*209,880*	*171,430*
NGA50	**TS**	0.21	1	n.d.	1
	**IS1**	1	0.998	1	0.98
	**IS2 or IS3**	0.36	0.78	0.74	0.75
	max**	*560,150*	*297,278*	*234,630*	*171,623*

* Relative assembly scores were rounded to the second decimal. In cases where a clear distinction between maximal and non-maximal scores would no longer be possible, rounding was extended to the first decimal place capable of being differentiated.

** *max* refers to the absolute value in nucleotides for the maximal relative assembly score of the column. *max* values represent averages that were rounded to nearest integer.

Table S13 – Relative assembly scores for factor read length (Edena assemblies)

quality metric	read length	genome Bce	Efa	Sen	Pst
corrected NG50	**100**	0.74	0.74	0.92	1
	**125**	0.99	0.83	0.98	0.99
	**150**	0.98	0.87	0.98	0.95
	**175**	0.98	1	1	0.81
	**189**	1	0.87	0.87	0.72
	max**	*299,749*	*317,133*	*195,108*	*173,950*
NGA50	**100**	0.67	0.74	0.87	0.999
	**125**	0.91	0.83	0.95	1
	**150**	0.90	0.87	0.98	0.94
	**175**	1	1	1	0.81
	**189**	0.97	0.87	0.80	0.72
	max**	*329,643*	*318,278*	*221,295*	*174,717*

* Relative assembly scores were rounded to the second decimal. In cases where a clear distinction between maximal and non-maximal scores would no longer be possible, rounding was extended to the first decimal place capable of being differentiated.

** *max* refers to the absolute value in nucleotides for the maximal relative assembly score of the column. *max* values represent averages that were rounded to nearest integer.

Table S14 – Relative assembly scores of factor sequencing depth (Edena assemblies)

quality metric	sequencing depth	genome* Bce	Efa	Pst
corrected NG50	**45**	0.58	0.57	0.57
	**90**	1	1	1
	max**	*355,661*	*348,269*	*197,405*
NGA50	**45**	0.57	0.58	0.57
	**90**	1	1	1
	max**	*374,324*	*348.271*	*198,796*

* Relative assembly scores were rounded to the second decimal.

** *max* refers to the absolute value in nucleotides for the maximal relative assembly score of the column. *max* values represent averages that were rounded to nearest integer.
