# Supplementary material for: Optimized Illumina PCR-free library preparation for bacterial whole genome sequencing and analysis of factors influencing de novo assembly
Source: BMC Res Notes. 2016 May 12;9:269. doi: 10.1186/s13104-016-2072-9 (PMC4864918; doi:10.1186/s13104-016-2072-9)

Additional file 2

Figure S1 – Intra-category GC-content dependent characteristics of average insert size

Average library insert sizes were estimated using BWA v0.6.2 (http://bio-bwa.sourceforge.net/) [1] and the Picard toolkit v1.84 (http://broadinstitute.github.io/picard/).

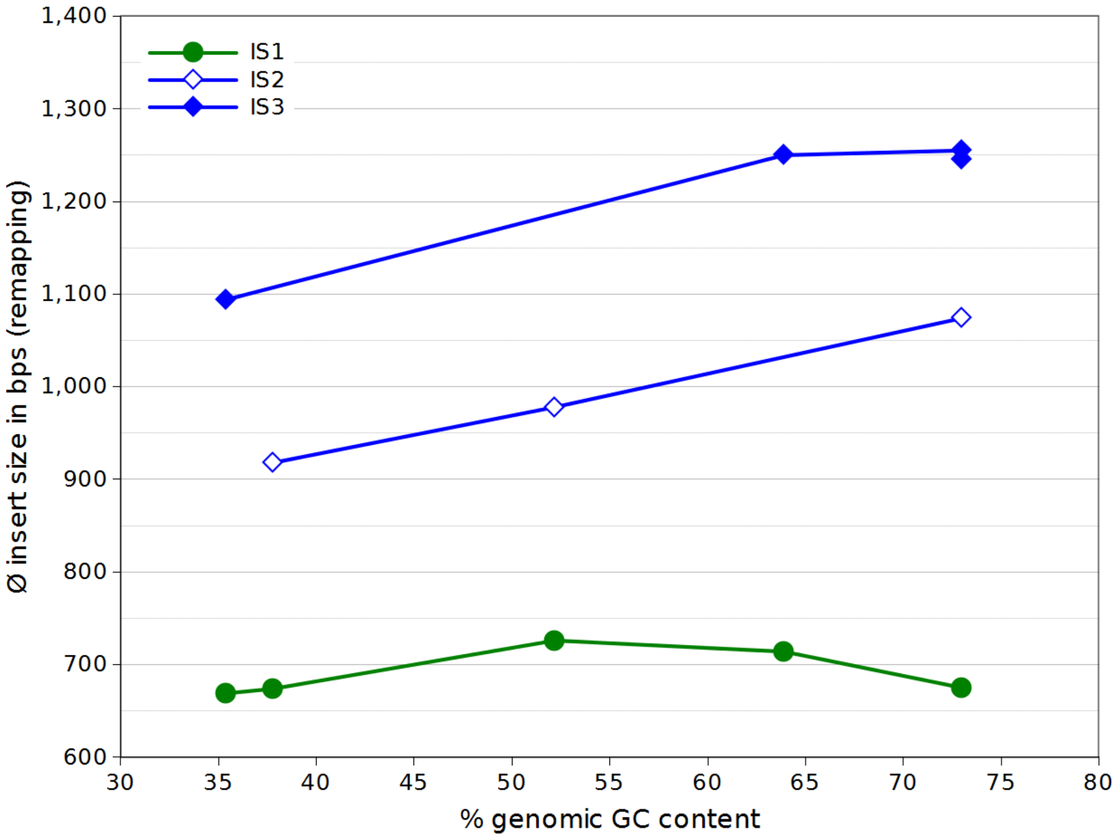

[1] Li H, Durbin R: **Fast and accurate long-read alignment with Burrows-Wheeler transform**. *Bioinformatics* 2010, **26**(5):589-595.

Figure S2 – Corrected NG50 plateau analysis of libraries with a read length of 189 nucleotides

Sequence assembly was performed with SPAdes v2.5.1 (http://bioinf.spbau.ru/spades) [1].

Underlying corrected NG50 values were determined with QUAST v2.2 (http://bioinf.spbau.ru/quast) [2].

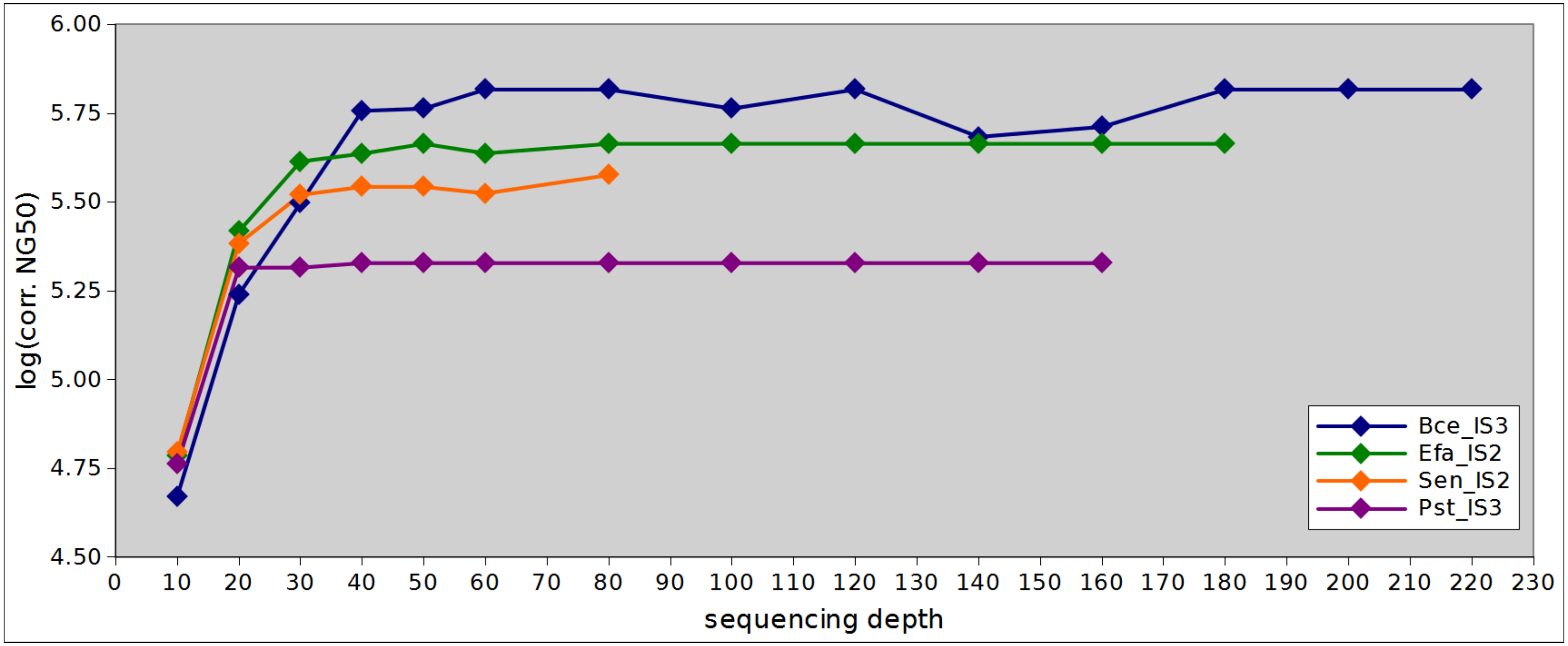

[1] Bankevich A, Nurk S, Antipov D, Gurevich AA, Dvorkin M, Kulikov AS, Lesin VM, Nikolenko SI, Pham S, Prjibelski AD *et al*: **SPAdes: a new genome assembly algorithm and its applications to single-cell sequencing**. *J Comput Biol* 2012, **19**(5):455-477.

[2] Gurevich A, Saveliev V, Vyahhi N, Tesler G: **QUAST: quality assessment tool for genome assemblies**. *Bioinformatics* 2013, **29**(8):1072-1075.

Figure S3 – NGA50 plateau analysis of libraries with a read length of 189 nucleotides

Sequence assembly was performed with SPAdes v2.5.1 (http://bioinf.spbau.ru/spades) [1].

Underlying corrected NG50 values were determined with QUAST v2.2 (http://bioinf.spbau.ru/quast) [2].

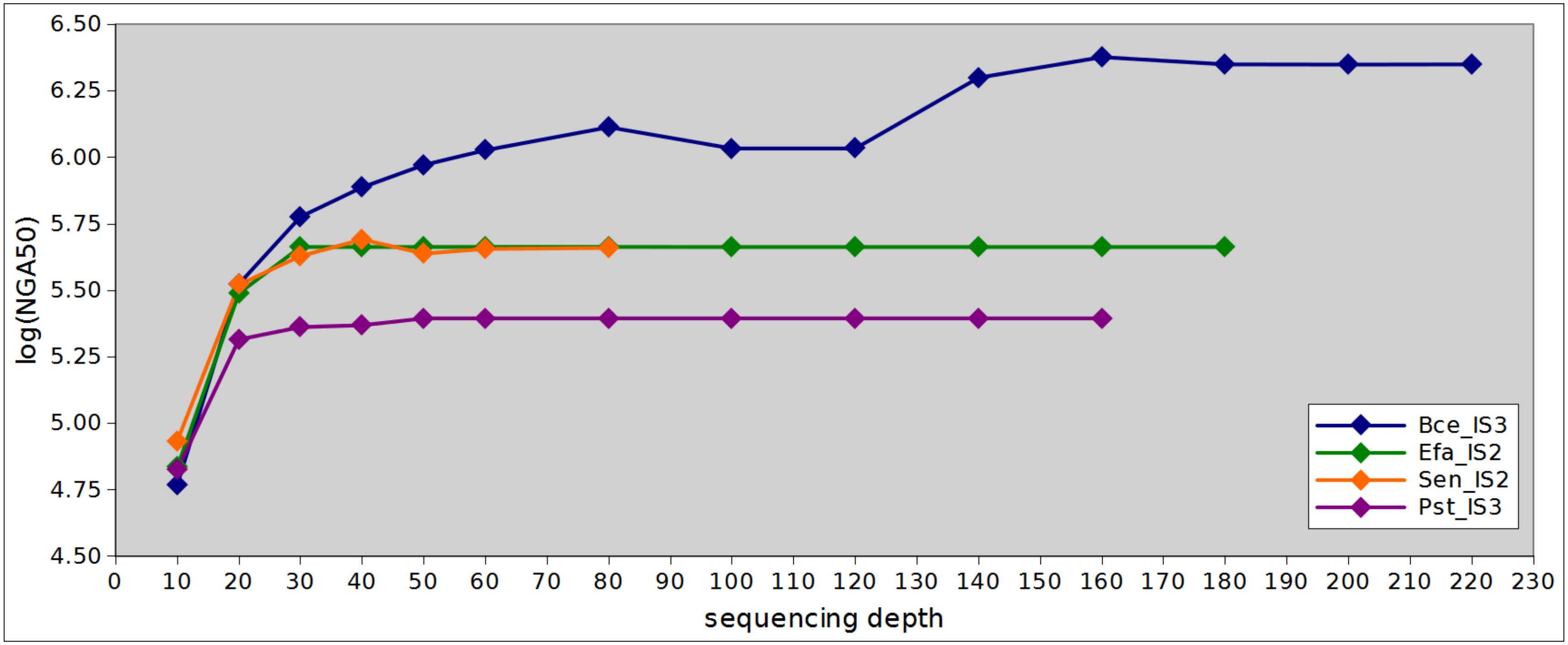

[1] Bankevich A, Nurk S, Antipov D, Gurevich AA, Dvorkin M, Kulikov AS, Lesin VM, Nikolenko SI, Pham S, Prjibelski AD *et al*: **SPAdes: a new genome assembly algorithm and its applications to single-cell sequencing**. *J Comput Biol* 2012, **19**(5):455-477.

[2] Gurevich A, Saveliev V, Vyahhi N, Tesler G: **QUAST: quality assessment tool for genome assemblies**. *Bioinformatics* 2013, **29**(8):1072-1075.

Figure S4 – Boxplot representation of Phred scores

Forward reads of quality filtered library Pst_IS1 are shown.

Analysis was performed with FastQC v0.10.1 (http://www.bioinformatics.babraham.ac.uk/projects/fastqc/).

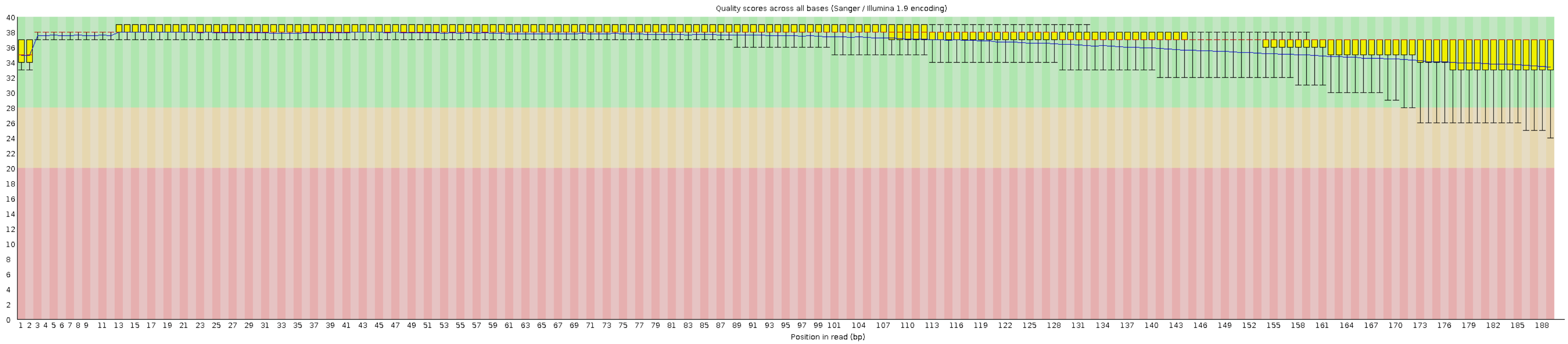

Figure S5 – FASTQC boxplot representation of Phred scores

Reverse reads of quality filtered library Pst_IS1 are shown.

Analysis was performed with FastQC v0.10.1 (http://www.bioinformatics.babraham.ac.uk/projects/fastqc/).

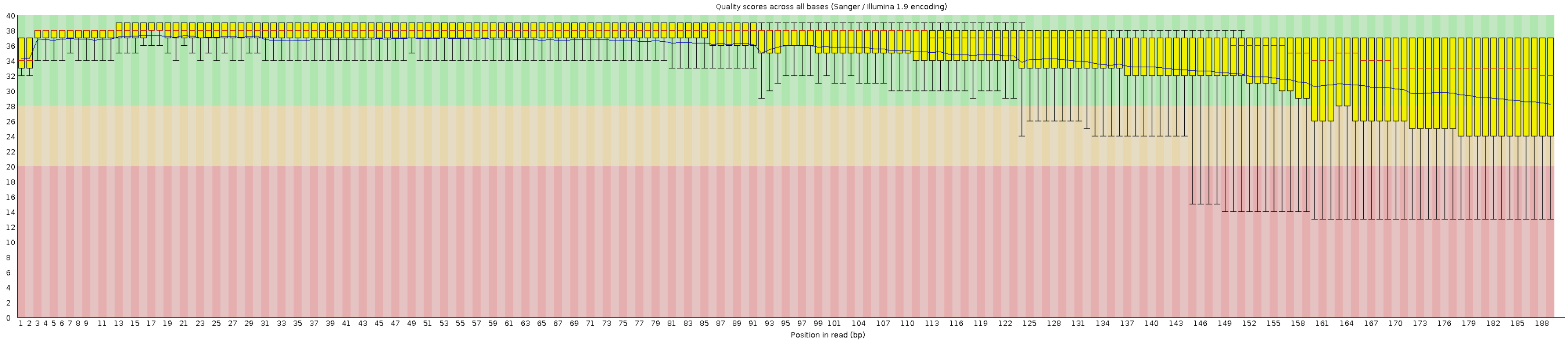

Supplement: Supplementary file 2 — 10.1186/s13104-016-2072-9 Intra-category GC-content dependent characteristics of average insert size. Figure S2. Corrected NG50 plateau analysis of libraries with a read length of 189 nucleotides. Figure S3. NGA50 plateau analysis of libraries with a read length of 189 nucleotides. Figure S4. Boxplot representation of Phred scores. Figure S5. FASTQC boxplot representation of Phred scores. [file 13104_2016_2072_MOESM2_ESM.pdf]
